# The Emergence of Hierarchical Structure in Human Language

**DOI:** 10.3389/fpsyg.2013.00071

**Published:** 2013-02-20

**Authors:** Shigeru Miyagawa, Robert C. Berwick, Kazuo Okanoya

**Affiliations:** ^1^Department of Linguistics and Philosophy, Massachusetts Institute of TechnologyCambridge, MA, USA; ^2^Department of Electrical Engineering and Computer Science and Laboratory for Information and Decision Systems, MITCambridge MA, USA; ^3^Department of Life Sciences, The University of TokyoTokyo, Japan

**Keywords:** human language, birdsong, honeybee, monkey communication, hierarchy

## Abstract

We propose a novel account for the emergence of human language syntax. Like many evolutionary innovations, language arose from the adventitious combination of two pre-existing, simpler systems that had been evolved for other functional tasks. The first system, Type E(xpression), is found in birdsong, where the same song marks territory, mating availability, and similar “expressive” functions. The second system, Type L(exical), has been suggestively found in non-human primate calls and in honeybee waggle dances, where it demarcates predicates with one or more “arguments,” such as combinations of calls in monkeys or compass headings set to sun position in honeybees. We show that human language syntax is composed of two layers that parallel these two independently evolved systems: an “E” layer resembling the Type E system of birdsong and an “L” layer providing words. The existence of the “E” and “L” layers can be confirmed using standard linguistic methodology. Each layer, E and L, when considered *separately*, is characterizable as a finite state system, as observed in several non-human species. When the two systems are put together they interact, yielding the unbounded, non-finite state, hierarchical structure that serves as the hallmark of full-fledged human language syntax. In this way, we account for the appearance of a novel function, language, within a conventional Darwinian framework, along with its apparently unique emergence in a single species.

## Introduction

Human language appears to be a recent evolutionary development, arising within the past 100,000 years, and has not evolved in any significant way since our ancestors left Africa, about 50,000–80,000 years ago (Tattersall, 2009). If so, the human language faculty emerged relatively suddenly in evolutionary time and has not evolved since.

How did this come about? While speculation about evolution without direct data remains challenging, it may still be possible to provide an account broadly compatible with what we know about human language syntax, along with the apparently rapid emergence of language. Contemporary human language syntax cannot be characterized by any finite state grammar (Chomsky, 1956). Such simple systems cannot properly represent the ambiguity found in human language, even for the simplest word strings such as *deep blue sky*. Finite state systems cannot represent such ambiguity because by definition they are *syntactic monoids*: algebraically, they must obey associativity, so they cannot assign two distinct representations to the concatenation *deep blue sky*. One needs a compositional operator that can take two lexical items, or in general any two syntactic objects, and assemble them into a single, newly labeled whole, beyond the power of any finite state grammar.

In this paper we advance a novel account for the emergence of this species-specific language property. Like many evolutionary innovations, we propose that language arose from the adventitious combination of two pre-existing, simpler systems evolved for other tasks. The first system, which we will call Type E, for *expressive*, can be found, for example, in birdsong (Berwick et al., 2011), where the same song serves to mark territory, mating availability, and other “expressive” functions. The second system, which we will call Type L, for *lexical*, has been suggestively observed in honeybees, where it demarcate a predicate with one or more “arguments” – here, elements of the honeybees’ dance corresponding to compass headings and flight paths (Riley et al., 2005). Somewhat controversial examples of Type L are monkey alarm calls that referentially convey types of predators (Seyfarth et al., 1980) and show combinatorial emergence of new semantics (Arnold and Zuberbuhler, 2006). Human language syntax integrates these two systems into single, composite, hierarchically structured whole by linking elements from the Lexical system with those of the Expressive system.

## A Simple Example Shows How to Link the E and L Systems

A simple question such as (1) illustrates how the Expressive and Lexical systems are linked.

**Table:** 

(1)	*What*_i_ do you think that Mary said John bought ____i_?

The phrase *what* occurs at the head of a sentence in an *expressive* “question position” but it is also semantically associated as a verbal argument in a *lexical* position after *buy* where it is given meaningful, interpretable content. The next section demonstrates that all such “linking relations” bridge the lexical and expressive layers, integrating the two systems.

## Human Language Sentences Contain Two Layers of Meaning

All human language sentences are composed of two meaning layers (e.g., Chomsky, 1995; Miyagawa, 2010). Consider (2).

**Table:** 

(2)	Did John eat pasta?

The core lexical meaning of (2) is formed from the words, *John, eat*, and *pasta*. Regardless of syntactic form, the *lexical meaning* fixed by these words remains intact: e.g., one can add modality or tense, as in, *John* may *eat pasta*; *John* will *eat pasta*. Separate from the lexical structure, sentence (2) contains the word *did*, which has two functions. The first expresses tense (*John* did *eat pasta*); the second expresses a question (did *John eat pasta*?). In this way, starting with lexical structure, Tense, and Question-formation output an expression that can be used in conversation. *Did* indicates a past event, and it forms a question about this event. This so-called “duality of semantics” (Chomsky, 2000) is represented as a hierarchical structure (Hale and Keyser, 1993; Chomsky, 2005; Miyagawa, 2010).

**Table:** 

(3)	Duality of semantics (Chomsky, 1995, 2000; Miyagawa, 2010)
	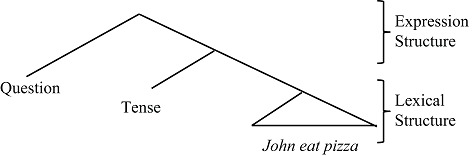

lexical structure is composed from a potentially open-ended set of lexical items that occur independently (*John, eat, pasta*). In contrast, expression structure is composed of limited number of elements typically characterized as “functional elements” that lack independent status, e.g., the past tense – *ed* in English (e.g., Hale and Keyser, 1993). As shown in (3), sentences are constructed with an “outer layer” of expression structure and an “inner layer” of lexical structure.

## Antecedents for Lexical Structure in Non-Human Animals

To make the case for an evolutionary precursor for lexical structure, one should locate in another animal species the ability to group two or three elements together, without syntax, arriving at an amalgamated “meaning.” In the honeybee waggle dance, the dance meaning may be decomposed into two parts, without syntax: dance direction conveys compass bearing for food location; dance speed conveys information regarding distance to a food source (Riley et al., 2005).

There is a large body of literature on the calls of monkeys and apes (Seed and Tomasello, 2010). Earlier studies concluded that Kenyan Vervet monkeys (Seyfarth et al., 1980) possess alarm calls for pythons, eagles, and leopards. In a sense, this is the simplest lexically based system where an uttered object correlates with a particular real-world state of affairs. More recently, there has been much debate as to whether non-human primates possess the ability to construe objects within an abstract event (Tomasello and Call, 1997). These studies suggest that non-human primate calls may be construed as lexical. For example, a number of studies have suggested that these primates perform reasonably well on Piagetian object permanence up to State 4 or 5 (Seed and Tomasello, 2010); they perceive objects even when they are no longer in their original location. There are even some recent studies in various primate species suggesting that these animals might use multiple calls to compose a novel meaning (Dessalles, 2007; Arnold and Zuberbuhler, 2008; Tallerman and Gibson, 2011).

## Birdsong and Expression Structure

**Figure 1:**
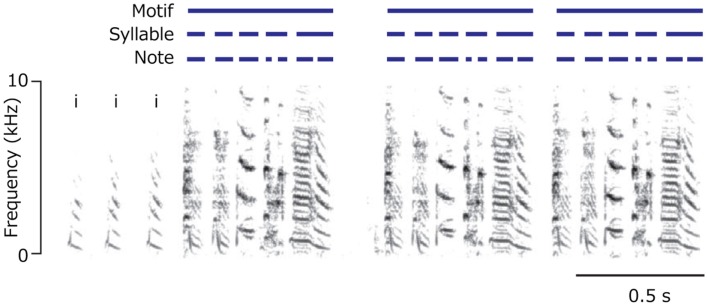
**Zebra finch song**.

Links between birdsong and human language have long been noted (Darwin, 1871; Jespersen, 1922; Marler, 1970; Nottebohm, 1975; Doupe and Kuhl, 1999; Okanoya, 2002; Bolhuis et al., 2010; Berwick et al., 2012). There are striking parallels between birdsong and human language acquisition: a need for external input; sensitive developmental periods ending at sexual maturity; hemispheric lateralization; and motor-auditory rehearsal systems (Bolhuis et al., 2010). Despite these similarities, what is striking about every variety of birdsong that has been studied is that lexical items in the sense of human languages remain absent (Berwick et al., 2011). Nor does birdsong contain the rich hierarchical structure characteristic of human language (Berwick et al., 2012). A typical case in point is the song of the zebra finch (Figure 1), which has a restricted set of “notes” that combine to form sequence of syllables, syllables into motifs, and motifs into complete song “bouts” (Berwick et al., 2011).

Other vocal learning bird species such as the Bengalese finch admit more complex patterns involving branches, loops, and repetitions.

**Figure 2:**
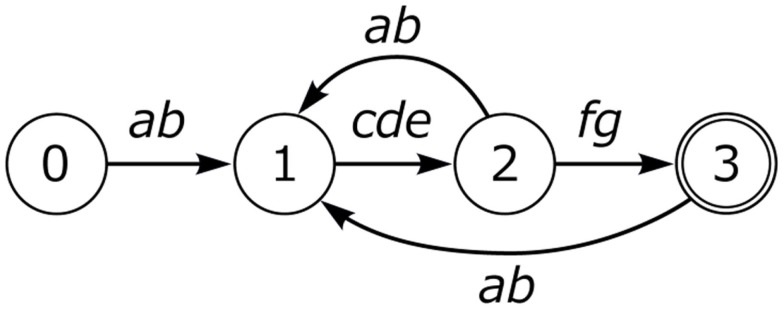
**Bengalese finch song**.

As shown in Figure 2, Bengalese finch song can loop to a preceding song position at various states, admitting considerable variation. Nightingales have an even more complex song structure, with possible branches at many more additional positions, with a single nightingale’s repertoire containing 100–200 distinct songs (Kipper et al., 2006). Nevertheless, all known birdsong examples can be described as a particular constrained kind of finite state automaton (Berwick et al., 2011).

There are two senses in which birdsongs lack lexical items, or “words.” First, song elements are never combined to yield new “meanings.” This is unlike primate calls mentioned above (Arnold and Zuberbuhler, 2008). Second, regardless of variety, birdsong conveys only a limited, holistic range of intentions, primarily related with reproduction. In this sense, birdsongs convey messages, not meanings (Tallerman and Gibson, 2011). We will refer to this type of language system as *Type E*, for *E*(*xpression*), without meaning.

## Birdsong and Human Language

Two items that Berwick et al. (2012) point out that human language has but birdsong does not are: (i) phrases “labeled” by element features (see below); (ii) hierarchical structure of phrases. These distinctions arise from the fact that human language possesses lexical items while birdsong does not. Thus, birdsong syntax is sometimes referred to as phonological syntax, emphasizing the lack of a lexicon (Marler, 2000).

Additionally, birdsong apparently lacks any “recursion,” in the sense that one bout can be hierarchically contained within another. We argue that just such a limitation is also imposed on human language, but only in the domain of expression structure, thereby drawing one key connection between birdsong and human language. Hence, the connection between birdsong and human language is not between song and language in its entirety; rather, the connection is between birdsong and the expression structure component of human language syntax. While it has been sometimes suggested that certain bird species can acquire recursive syntactic structures either through conditioning (Gentner et al., 2006) or spontaneously (Abe and Watanabe, 2011), this result remains controversial and, as noted in Beckers et al. (2012), so far unconfirmed. Nonetheless, it seems plausible that some abilities for processing temporally ordered acoustic streams are shared by both avian and human vocal learners. By necessity, sound streams must be parsed into beginning and ending “chunks” – words and word components in the case of humans, or syllable chunks for songbirds. Without word boundaries and word pattern recognition, human language acquisition becomes impossible; this is clearly required for early vocal learning. The same holds for birds and syllable chunks (Takahasi et al., 2010).

**Table:** 

(4)	Human language and the non-human language-like types
	LEXICAL STRUCTURE ↔ [BEES/PRIMATES] TYPE L
	EXPRESSION STRUCTURE ↔ [BIRDSONG] TYPE E

## Labeling

A second unique feature of human language is “labeling” (Chomsky, 1995). Given a word, its category (Noun, Verb, etc.) forms the label of the larger phrase that contains it. For instance, given the pair *eat* and *the apples*, the verb *eat* labels the larger phrase, *eat the apples* (conventionally, a Verb Phrase).

**Table:** 

(5)	Labeling
	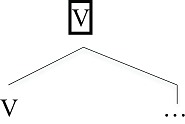

In this way, phrases in human language have the same property as the original lexical item that provided the label (Chomsky, 1995, 2008; Hornstein, 2009). This gives human language its unique ability to form hierarchical structures (Chomsky, 1995, 2008; Hornstein, 2009), as we now detail.

### Expression structure: Limited hierarchy and labeling

The labeling phenomenon above appears to be uniquely human. It occurs with all kinds of phrases (e.g., Noun, Verb, Preposition) such that human syntactic structure has the property of “discrete infinity” (Chomsky, 2000) through recursively merging and labeling structures. However, on close examination, there is a severe limitation on the depth of the hierarchy for one component of human language. Recall that expression structure can contain an item with property Tense; there is a second item, conventionally labeled “C(omplementizer)” that hosts a range of expressive phrases such as Q(uestion), F(ocus) (e.g., *Starlings, I like*), and so forth, as shown in (6).

**Table:** 

(6)	Expression Structure
	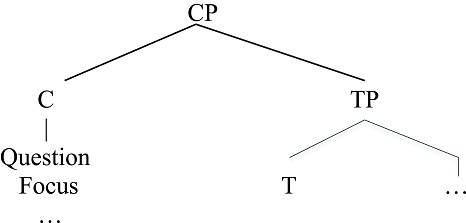

These are the two most frequently cited “labels” within the expression structure. Strikingly, these labels cannot be assembled as hierarchical structures of arbitrary depth. Rather, the CP-TP hierarchical structure can be only one layer deep, as in (6). Predictably, one does not find human language hierarchical structures such as the following (the asterisk marks an impossible form). Such unattested structures would correspond to having successive layers of Question or Focus phrases, for example (Arsenijevic and Hinzen (2012) make a similar observation based on meaning).

**Table:** 

(7)	An impossible syntactic structure
	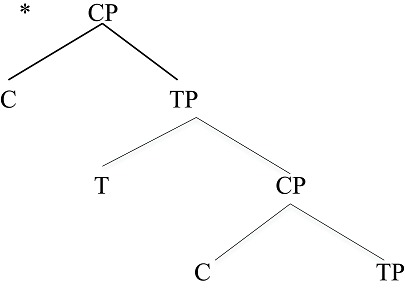

This is similar to the restriction noted earlier for birdsong: here we also find a depth-one hierarchical structure, as with the Bengalese finch and Nightingale songs. This suggests that expression structure itself is of Type E, closely reflecting birdsong structure. Importantly then, there is no recursion through the CP-TP expression structure. There are theories of ES (Rizzi, 1997) that posit a multi-layer within Expression Structure to deal with such phenomena as topicalization and focus. However, there are alternatives that do not assume such a multi-layer (e.g., Miyagawa, 2010). Phenomena such as prosody map onto these hierarchical structures.

### Lexical Structure

Unlike expression structure, lexical structure elements cannot directly combine with each other to form purely LS hierarchical structures:

**Table:** 

(8)	Examples of impossible lexical structures
	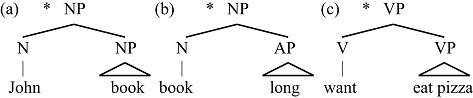

To make (8a) possible, for example, one must insert *s* (*John*’S *book*); this *s* is D(eterminer; Abney, 1987), a member of the expression structure (see below). The fact that L does not allow hierarchy matches the constraints on type l languages found in non-human primates and bees.

## The Source of Discrete Infinity

If ES only admits one layer of hierarchical structure and LS does not admit any hierarchical structure, what is the source of human syntax’s unbounded hierarchical structure? The answer lies in the way unbounded hierarchical structures are assembled, typically combinations that interweave the E and L levels:

**Table:** 

(9)	E/L hierarchical structure (“D(eterminer)” is part of the ES for noun phrases)
	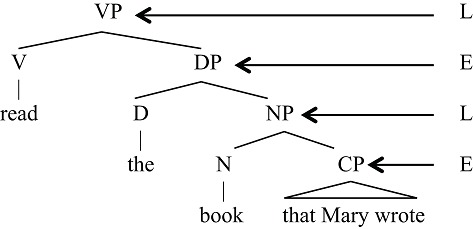

In this fragment of a sentence, we see an alternation of L and E structure. One can abbreviate this in the form of conventional context-free re-write rule as follows, where EP is the “label” for a category of the type E and “LP” is the “label” for an L-type structure.

**Table:** 

(10)	(i) EP → E LP
	(ii) LP → L EP

Rule (i) states that the E category can combine with LP to form an E-level structure. Rule (ii) states that the L category can combine with an E-level structure to form an L-level structure. Together, these two rules suffice to yield arbitrarily deep hierarchical structures. If we expand the left-hand side of Rule (i), EP, we obtain the two items on the right-hand side of the rule, E LP. We may enclose these with square brackets, to indicate that they form a complete EP phrase [E LP]. Now we can apply Rule (ii) to LP, expanding it as, L EP. Again using bracket notation, we obtain the form [E [L EP]]. We can once more apply Rule (i) to the EP unit that is now embedded within the brackets, obtaining [E [L [E LP]]], and continue this *ad infinitum* to yield arbitrarily nested hierarchical structure. All current empirically adequate linguistic theories contain some means like this to build such kinds of structures. Arbitrarily deep hierarchical structure is thus the by-product of E- and L-structures combining alternately. Each component by itself is describable by a finite state grammar. However, when combined, they interact to yield the familiar arbitrarily deep hierarchical structure we associate with human language.

**Table:** 

(11)	ES: finite state
	LS: finite state

## E/L Integration Hypothesis

Given the difference between expression structure and lexical structure, we propose that human language arose by *integrating* these two distinct systems, type l (lexical) and type e (expression):

**Table:** 

(12)	Integration of E and L
	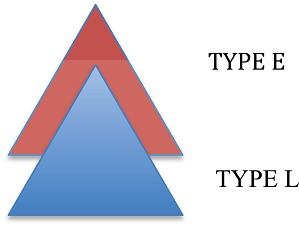

How does this integration work? Two properties found in human languages that have been the focus of intensive study in linguistics, displacement and agreement, have in common the property that they link an item from one layer with an item from the other layer (Miyagawa, 2010), thereby uniting the two layers. We discuss displacement below.

### From lexical structure to expression structure

The displacement of labeled phrases always occurs from lexical structure to expression structure. In forming English questions, some question word that first occurs in the lexical structure is displaced to the C position in the expression structure.

**Table:** 

(13)	What did you eat ___ ?

In Chinese, to indicate the topic of a sentence, a similar displacement occurs

**Table:** 

(14)	Zheben shu Zhangsan mai-le ___.
	this book Zhangsna buy-ASPECT
	‘This book, Zhangsan bought.’

We can picture this displacement as follows:

**Table:** 

(15)	Displacement from L to E
	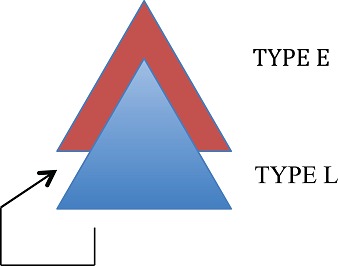

By displacing an item from the lexical structure to the expression structure, these two layers of language are then linked. We therefore posit the following principle (16).

**Table:** 

(16)	Displacement exists to integrate the Expression and Lexical structures of human language.

## Conclusion and Directions for Future Inquiry

Our proposal partitions language syntax into two systems, E and L, locating suggestive antecedents for each in non-human animals. We have outlined how these two systems could be integrated to yield the discrete infinity of human language. How did the E and L systems come to be linked in modern humans? While answers to this question must necessarily remain speculative, one can advance at least two possible routes. One involves shared human intentionality (Tomasello et al., 2005). Although there is limited evidence that alarm calls in monkeys are under intentional control (Seyfarth and Cheney, 2010), this ability appears full-blown in humans. Shared intentionality adds an expressive component to the lexical system, in this way functionally interleaving the E and L systems. A second possibility is the one noted by Darwin (1871) in his *Descent of Man*: human language first emerged as “songs” – prosodic contours and syllable structures like birdsong – which were then grafted onto a separate word system. In this article we have attempted to advance Darwin’s hypothesis. (Others have embraced Darwin’s proposal, though without our division into E and L systems; see, e.g., Fitch (2010).) Additionally, the ability to “chunk” acoustic streams into linear segments, along with prosody or metrical structure – the pattern of strong and light “beats” in a song – rhythmic entrainment, and vocal learning, are shared among vocal learning avian species as well as humans. While neurobiology points to right-brained localization for human prosodic processing, it is well known that syntactic processing is localized to left-brain areas in humans, while “naming” involves both dorsal and ventral streams (Friederici, 2012). Taken together, one might speculate, following Berwick (2011), that the purely finite system for metrical structure – a right-brain activity – was joined with the “naming” ability of early humans (or possibly other primates) to yield the combination E-L system and so fully human language.

## Conflict of Interest Statement

The authors declare that the research was conducted in the absence of any commercial or financial relationships that could be construed as a potential conflict of interest.
